# Investigating the potential of Shikonin as a novel hypertrophic scar treatment

**DOI:** 10.1186/s12929-015-0172-9

**Published:** 2015-08-16

**Authors:** Chen Fan, Yan Xie, Ying Dong, Yonghua Su, Zee Upton

**Affiliations:** Tissue Repair and Regeneration Program, Institute of Health and Biomedical Innovation, Queensland University of Technology, Brisbane, Queensland 4059 Australia; Tissue Organ Bank & Tissue Engineering Centre, General Hospital of Ningxia Medical University, Yinchuan, China; Cancer Research Program, Translational Research Institute, Queensland University of Technology, Brisbane, Australia; Changhai Hospital of Traditional Chinese Medicine, Second Military Medical University, Shanghai, China

## Abstract

**Background:**

Hypertrophic scarring is a highly prevalent condition clinically and results from a decreased number of apoptotic fibroblasts and over-abundant production of collagen during scar formation following wound healing. Our previous studies indicated that Shikonin, an active component extracted from *Radix Arnebiae,* induces apoptosis and reduces collagen production in hypertrophic scar-derived fibroblasts. In the study reported here, we further evaluate the potential use of Shikonin as a novel scar remediation therapy by examining the effects of Shikonin on both keratinocytes and fibroblasts using Transwell® co-culture techniques. The underlying mechanisms were also revealed. In addition, effects of Shikonin on the expression of cytokines in Transwell co-culture “conditioned” medium were investigated.

**Results:**

Our results indicate that Shikonin preferentially inhibits cell proliferation and induces apoptosis in fibroblasts without affecting keratinocyte function. In addition, we found that the proliferation-inhibiting and apoptosis-inducing abilities of SHI might be triggered via MAPK and Bcl-2/Caspase 3 signalling pathways. Furthermore, SHI has been found to attenuate the expression of TGF-β1 in Transwell co-cultured “conditioned” medium.

**Conclusions:**

The data generated from this study provides further evidence that supports the potential use of Shikonin as a novel scar remediation therapy.

## Background

Hypertrophic scarring (HS) is a highly prevalent condition that occurs after burns and surgical incision [1]. Pain, itching, stiffness, loss of sensation and loss of joint mobility have been reported by patients with HS [2]. Although the exact mechanisms underpinning HS formation are still elusive, the formation of HS results from the dysregulation of the wound healing process [3]. When sufficient collagen is formed at the end of wound healing, the number of apoptotic fibroblasts sharply increases. In HS, however, large amounts of fibroblasts persist, producing an over-abundance of collagen [4, 5]. In addition, delayed keratinocyte (Kc) function in terms of re-epithelialization also leads to HS formation [6]. Furthermore, exaggerated inflammation may lead to HS formation by prolonging the wound healing process [7].

Current scar-remediation therapies are less than satisfactory for a number of reasons [8], hence attention has been given to the potential benefits of natural products as an alternative strategy to remediate scars [9]. Shikonin (SHI), an active component extracted from the Chinese herb *Radix Arnebiae*, has been widely demonstrated to possess various biological activities, such as anti-inflammatory, anti-bacterial, anti-angiogenic and anti-tumorigenic properties [10]. Most importantly, SHI has been extensively reported to induce apoptosis in many different cancer cell lines [11, 12]. Based on the importance of apoptosis in HS formation and the apoptosis-inducing ability of SHI, we therefore investigated the effects of SHI on hypertrophic scar-derived human skin fibroblasts (HSF). Our preliminary studies indicated that SHI reduces HSF proliferation and collagen production in a dose-dependent manner and the underlying mechanisms have also been partly revealed (Article in press).

Given that wound healing is a complex process that requires the participation of different types of cells, such as Kc and fibroblasts, we have now progressed to evaluating the effects of SHI on both Kc and HSF using the Transwell® co-culture technique and investigated the underlying molecular mechanisms. In addition, considerable evidence indicates that crosstalk between Kc and fibroblasts plays an essential role in both wound healing and HS formation. This crosstalk is mediated by soluble cytokines and growth factors, rather than by direct interaction [13]. Thus, the paracrine effects of SHI on cytokines in Kc and fibroblast were also investigated.

## Methods

### Preparation of reagents

SHI powder was produced by the National Institute for the Control of Pharmaceutical and Biological Products, China. SHI was dissolved in Dimethyl sulfoxide (DMSO; Sigma-Aldrich, Australia) as a stock solution and stored at −20 °C before use. Phospho-ERK inhibitor U0126 (#9903) and Phospho-JNK inhibitor SP600125 (#8177) were purchased from Cell Signalling, Australia. U0126 and SP600125 were diluted into DMSO at 10 μM and 50 μM before use.

### Cell culture

Primary human Kc were isolated from native human skin obtained from consenting donors at St Andrew’s Hospital (Brisbane, Australia) with human ethics approval from both the hospital (2003/46) and the Queensland University of Technology (1300000063). Kc were cultured in “Green’s” medium containing 10 % fetal calf serum (FCS; Hyclone, Australia) following methods previously described by Rheinwald and Green (1975) and others [14, 15]. HSF were purchased from Cell Research Corporation (Singapore). These cells were routinely cultured in Dulbecco’s Modified Eagle’s Medium (DMEM; Invitrogen, Australia) containing 10 % FCS at 37 °C in an incubator with 5 % CO_2_.

### Cell proliferation assay

The effects of SHI on cell proliferation were investigated using the CyQUANT assay (Invitrogen). Kc (4 × 10^4^ cells/well) and HSF (6 × 10^4^ cells/well) were seeded into the 12-well Transwell® (Kc in the insert and HSF in the lower chamber, Corning, USA) for 48 h based on previously optimized cell density studies. Serial dilutions of SHI were then applied to the Transwell® resulting in final concentrations of 0.5, 1 and 3 μg/mL. A group without SHI treatment was also included as a control. After 72 h incubation, the CyQUANT reagents were applied as per the manufacturer’s instructions and cell proliferation was measured at λ_ex_ 485P, λ_em_ 520P using a Polar Star Optima Microplate Reader (Optima, Germany).

### Terminal deoxynucleotidyl transferase dUTP nick end labeling (TUNEL) assay

Apoptosis was detected using the TUNEL assay (Roche Applied Science, Australia) [16]. Kc and HSF were seeded and treated with SHI as described above. Following 48 h of incubation at 37 °C, the cells were first fixed in 3.7 % para-formaldehyde and then permeabilised in 0.2 % Triton X-100/PBS (Sigma-Aldrich). After incubation with the TUNEL reaction mixture for 1 h at 37 °C, a nuclear stain (4′, 6-diamidino-2-phenylindole (DAPI)) was added. Fluorescence images were then captured using a Nikon Eclipse TE2000-U microscope (Nikon, Australia).

### Flow cytometry

Flow cytometry was also used to indentify SHI-induced apoptosis in Kc and HSF using the Dead Cell Apoptosis Kit with Alexa® Fluor 488 annexin V and PI assay kit (V13241, Life Technologies). Phosphatidyl serine (PS) is normally located on the cytoplasmic surface of the cell membrane, whereas it translocates from the inner to the outer leaflet of the membrane when apoptosis occurs [17]. Annexin V is the protein that binds with PS, therefore apoptosis can be identified using fluorophore-labeled Annexin V. Briefly, Kc and HSF were treated as described above. The cells were harvested after 12 h of treatment, followed by washing with cold PBS twice. The cells were then resuspended in annexin-binding buffer at 2 × 10^5^ cells/mL and stained with Alexa® Fluor 488 annexin V and propidium iodide (PI) for 15 min at room temperature. The florescence was then measured at 530–575 nm emission and 488 nm excitation using FACSAria™ III Cell Sorter (Becton Dickinson, USA).

### Western blot

SHI-induced changes in protein expression were determined by Western Blot. Kc and HSF were first seeded and treated with SHI. Whole lysates from cells, excluding medium, were collected in a lysis buffer containing 150 mM NaCl, 50 mM Tris, 1 % sodium dodecyl sulphate, 1 % Triton, protease inhibitor cocktail (Roche Applied Science), 2 mM sodium vanadate and 10 mM sodium fluoride after 24 and 48 h of exposure to SHI. The protein concentrations were measured using the Bicinchoninic Acid (BCA; Pierce, USA) assay. Equal amounts of protein in each group were prepared and separated using 12 % sodium dodecyl sulphate polyacrylamide gel electrophoresis (SDS-PAGE) and were then transferred onto nitrocellulose membranes (Bio Rad, USA). The membranes were incubated with primary antibodies overnight at 4 °C in Odyssey blocking buffer (LI-COR® Biosciences, USA). Primary antibodies included: ERK1/2, p-ERK1/2, JNK1/2, p-JNK1/2, Caspase 3, Bcl-2, NF-κB, p-NF-κB, I-κB, p-I-κB, IKK-α/β and p-IKK-α/β from Genesearch, Australia; p38α/β and p-p38α/β from Santa Cruz Biotechnology, USA; and GAPDH from Sigma-Aldrich. Secondary antibodies conjugated with AlexaFluor 680 or 800 (Invitrogen) were then applied as species appropriate. Images were captured and analysed using the Odyssey Infrared Imaging system and software (LI-COR® Biosciences).

### Quantitative reverse transcriptase polymerase chain reaction (qRT-PCR)

qRT-PCR was used to evaluate the expression of genes in cells treated with SHI. Genes of interest and their respective primers are listed in Table 1. The cells were seeded into Transwells and treated with SHI for 24 h. Total RNA was then extracted from the cells using the Qiagen RNeasy Mini kit (Qiagen, Australia), as per the manufacturer’s protocol. First strand cDNA synthesis was performed using SuperscriptTM III Reverse Transcriptase (Invitrogen). qRT-PCR was then performed using the SYBR Green method in an ABI 7500 Thermal Cycler (Applied Biosystems, Australia).

**Table 1:** Primers used in qRT-PCR

Protein name	Corresponding gene name	Primers: Forward (F) & Reverse (R)
Caspase 3	*CASP3*	F: 5’-CGGAAGCAGTGCAGACGCGG-3’
		R: 5’-GCTGCGAGCACTCACGAAACTCTTC-3’
Bcl-2	*BCL2*	F: 5’-TCAACCGGGAGATGTCGCCCC-3’
		R: 5’-ACAAAGGCATCCCAGCCTCCGTT-3’
Bax	*BAX*	F: 5’-AAACTGGTGCTCAAGGCCC-3’
		R: 5’-TGAGGAGTCTCACCCAACCA-3’
Cytochrome *c*	*CYCS*	F: 5’-ATGGTCTCTTTGGGCGGAAG-3’
		R: 5’-CTCCCCAGATGATGCCTTTGT-3’
Collagen I	*COL1A1*	F: 5’-ACGAAGACATCCCACCAATC-3’
		R: 5’-AGATCACGTCATCGCACAAC-3’
Collagen III	*COL3A1*	F: 5’-GCCTCCCGGAAGTCAAGGAGAAAG-3’
		R: 5’-CTTTAGGACCGGGGAAGCCCATG-3’
αSMA	*αSMA*	F: 5’-CTGCTGAGCGTGAGATTGTC-3’
		R: 5’-CTCAAGGGAGGATGAGGATG-3’
GAPDH	*GAPDH*	F:5’-TCTTTTGCGTCGCCAGCCGAG-3’
		R:5’-TGACCAGGCGCCCAATACGAC-3’

### Enzyme-linked immunosorbent assay (ELISA)

Expression of transforming growth factor beta one (TGF-β1) in Transwell “conditioned” media was determined using ELISA assay kits (Invitrogen). Briefly, media in the Transwell® were collected at 24 and 48 h following SHI treatment. The ELISA assay was then performed as per the manufacturer’s instructions. Expression of TGF-β1 was measured at 450 nm using a Microplate Reader (Optima).

### Statistical analysis

Triplicate samples were assayed in each experiment and each experiment was replicated three times, each time using cells obtained from three different patients. The data obtained in each experiment were first converted to the percentage of the untreated control, and then the converted data from 3 different patients were pooled together as the final average data. One-way ANOVA and Tukey’s post-hoc test were applied and *p* < 0.05 was considered to be statistically significant.

## Results

### SHI inhibits cell proliferation and induces apoptosis in Kc and HSF

**Fig. 1:**
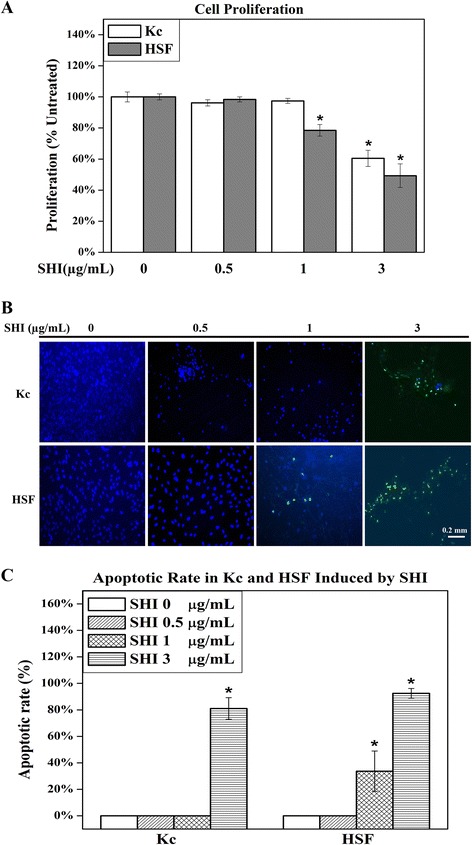
Effects of SHI on cell proliferation and apoptosis. **a** Cell proliferation. Kc and HSF were co-cultured and treated with SHI (0.5, 1 and 3 μg/mL) for 72 h. Cell proliferation was measured using the CyQUANT assay. The data were expressed as the average percentage of the untreated control (0 μg/mL SHI) containing DMEM medium alone for 72 h and were pooled from the average data from five replicate experiments (with cells from 5 different patients) in which each treatment was tested independently in triplicate. Error bars indicate mean +/− SEM (*n* = 5). *p < 0.05 versus the untreated control. Statistical analysis was performed using One-way ANOVA and Tukey’s post-hoc test. **b** Representative images showing apoptosis induced by SHI treatment in Kc and HSF respectively using TUNEL assay. Kc and HSF were treated with SHI for 72 h at the concentrations indicated, and then stained with TUNEL reagent to detect apoptotic cells and DAPI to detect the nuclei of all cells. Cells were viewed and images were captured using a Nikon Eclipse TE2000-U system. Green indicates DNA fragments from apoptotic cells, whereas blue localises the nuclei of both live and apoptotic cells. Representative images from cells obtained from three patients are depicted. Scale bar: 0.2 mm. **c** Apoptotic rate (%) in Kc and HSF induced by SHI. Three randomly selected images were recorded and the numbers of green-staining apoptotic cells and blue nuclei for all cells were counted. The final data was the average cell number of nine different images from three different patients. Apoptotic rate = number of green cells / (number of green cells + number of blue cells). Error bars indicate SEM

To identify the effects of SHI on cell proliferation, Kc and HSF were treated with different concentrations of SHI (0.5, 1 and 3 μg/mL) (Fig. 1a). SHI at 0.5 μg/mL showed no significant effects on both Kc and HSF proliferation compared to the untreated control (*p* < 0.05). SHI at 1 μg/mL decreased HSF proliferation by 21.5 % ± 3.7 % compared to the untreated control (*p* < 0.05), however, no inhibitory effect on Kc proliferation was detected at this concentration of SHI. Kc and HSF proliferation were 40.5 % ± 5.2 % and 50.7 % ± 7.6 % below the untreated control (*p* < 0.05) when exposed at SHI 3 μg/mL, respectively. Taken together, these data indicate SHI reduces both Kc and HSF proliferation in a dose-dependent manner with higher concentrations required, however, to elicit effects on Kc compared to HSF. Specially, 1 μg/mL SHI inhibits HSF but not Kc proliferation.

DNA fragmentation is an important hallmark indicating apoptosis and can be detected through the addition of TUNEL reagent, resulting in green emissions [16]. Observation of images captured of the cells when viewed with fluorescence microscopy (Fig. 1b) reveals that no apoptotic cells (green emissions) were detected in either Kc or HSF when treated with SHI 0.5 μg/mL. Apoptotic cells were observed in HSF but not in Kc when exposed to SHI at 1 μg/mL. SHI at 3 μg/mL, however, induced apoptosis in both Kc and HSF. Quantitative analysis of the TUNEL assay images revealed that SHI at 3 μg/mL induces 81.0 % ± 8.1 % and 92.5 % ± 3.7 % of the cells present to undergo apoptosis in Kc and HSF, respectively (Fig. 1c). Further, SHI at 1 μg/mL triggers 33.7 % ± 15.2 % of the HSF to undergo apoptosis. These results demonstrate that SHI induces apoptosis in both Kc and HSF in a dose-dependent manner and that Kc are more resistant to SHI-induced apoptosis than HSF.

**Fig. 2:**
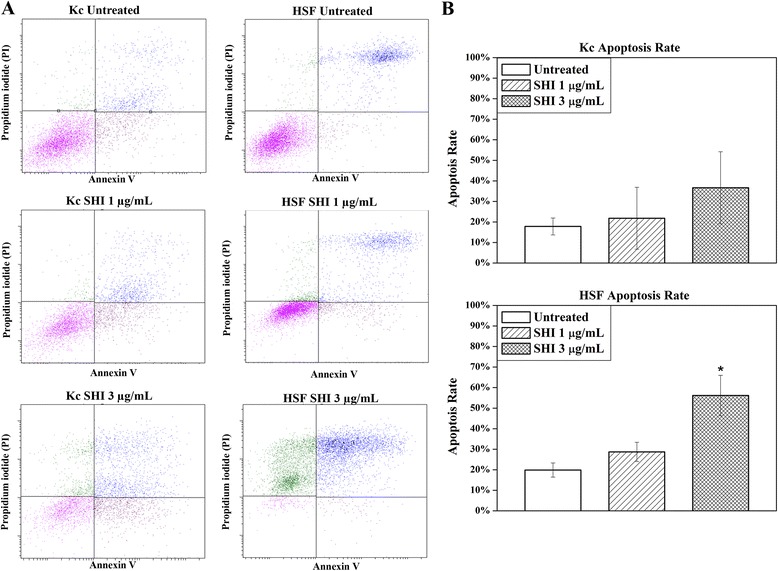
Apoptosis in Kc and HSF determined by flow cytometry. **a** Apoptosis rate in Kc and HSF following SHI treatment; **b** Quantitative analysis of SHI-induced apoptosis in Kc and HSF. Kc and HSF were treated with 0, 1 or 3 μg/mL SHI for 12 h and then stained with annexin V and propidium iodide as per the manufacturer’s instructions. Flow cytometry was performed using FACSAria™ III Cell Sorter (Becton Dickinson). All experiments were performed three times using cells from 3 patients. Triplicate treatments were assessed in cells from each patient. Quantitative data from the 3 patients were pooled. *p < 0.05 versus the untreated control

Data from flow cytometry (Fig. 2a & b) indicated that SHI at either 1 or 3 μg/mL showed no effect on Kc apoptosis compared to the untreated group. However, SHI at 3 μg/mL significantly induced 56.16 % ± 9.85 % HSF apoptosis at 12 h compared to the untreated control (*p* < 0.05). This data suggests that SHI at 3 μg/mL only induces apoptosis in HSF but not in Kc after 12 h of treatment.

### Effects of SHI on MAPK and intrinsic apoptosis signalling pathways

**Fig. 3:**
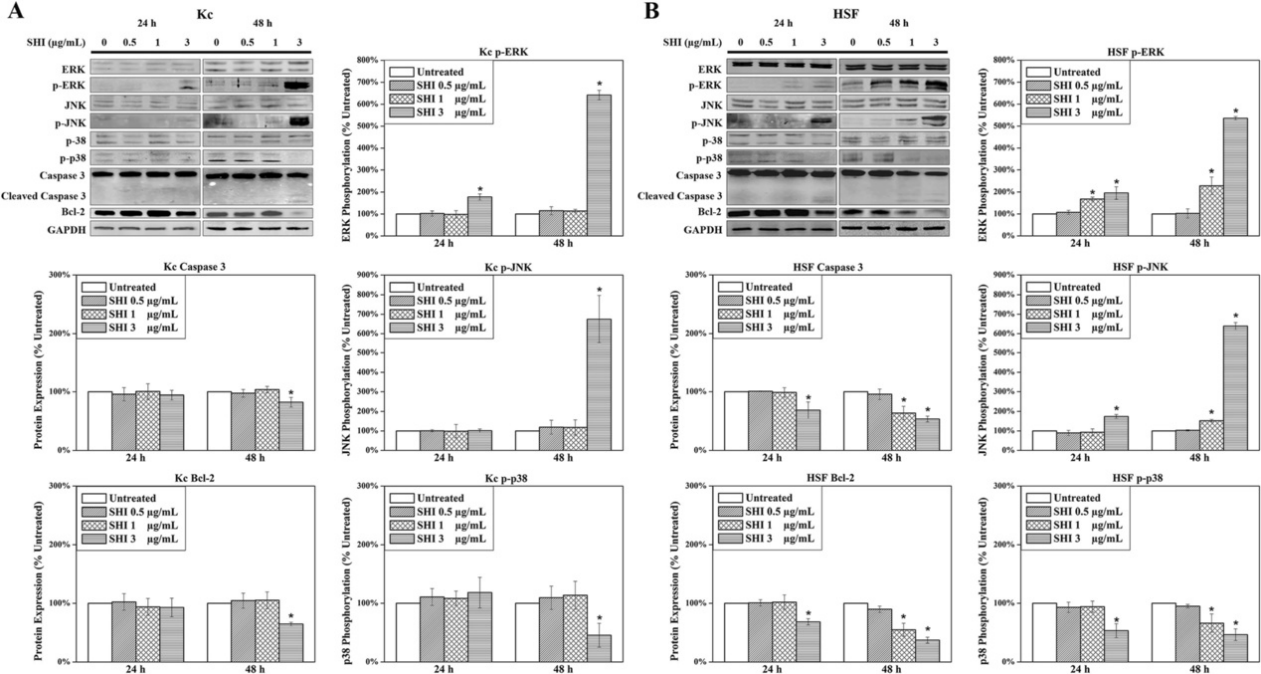
Effects of SHI on cell protein expression. **a** Protein Expression in Kc; **b** Protein expression in HSF. Proteins were collected separately from Kc and HSF treated with SHI for 24 and 48 h. The expression of proteins was detected using the Odyssey Infrared Imaging system. GAPDH was included as a loading control. For quantitative analysis, the intensities of the bands were measured with densitometry and first normalized to GAPDH and then further converted to the percentage of the untreated control. The converted data from 5 different patients were pooled together as shown in the figure. Representative images of the western blots are presented. Error bars indicate mean +/− SEM (*n* = 5). *p < 0.05 versus the untreated control. Statistical analysis was performed using One-way ANOVA and Tukey’s post-hoc test

Mechanisms underlying the proliferation-inhibiting and apoptosis-inducing ability of SHI were determined using western blot approaches (Fig. 3). Mitogen-activated protein kinases (MAPK), including ERK1/2, JNK1/2 and p38α/β, have been widely demonstrated to play essential roles in cell proliferation and apoptosis [18]. Those kinases are activated by phosphorylation [19]. In addition, Bcl-2 can indirectly cleave caspase 3 by releasing cytochrome *c* from the mitochondria [20]. Cleaved caspase 3 will then further induce apoptosis [21]. As shown in Fig. 3a & b, increases in phosphorylated ERK1/2 and JNK1/2 (p-ERK1/2, p-JNK1/2), decreases in phosphorylated p38α/β (p-p38α/β) and Bcl-2, as well as cleavage of caspase 3, were observed in Kc at 48 h and in HSF at either 24 or 48 h after exposure to 3 μg/mL SHI compared to the untreated control (*p* < 0.05). These changes were also found in HSF when treated with 1 μg/mL SHI for 48 h, but no effect on Kc protein expression was detected at this dose of SHI. Taken together these results indicate that SHI increases p-ERK1/2 and p-JNK1/2, decreases p38α/β and Bcl-2 and induces cleavage of caspase 3 in both Kc and HSF in a dose-dependent manner. Again, these changes in protein expression occur more rapidly (24 h) in HSF than Kc at the same dose of SHI (3 μg/mL). SHI at 1 μg/mL can also trigger these changes in HSF but does not induce changes in these proteins in Kc.

### Link between MAPK and intrinsic apoptosis signalling pathway

**Fig. 4:**
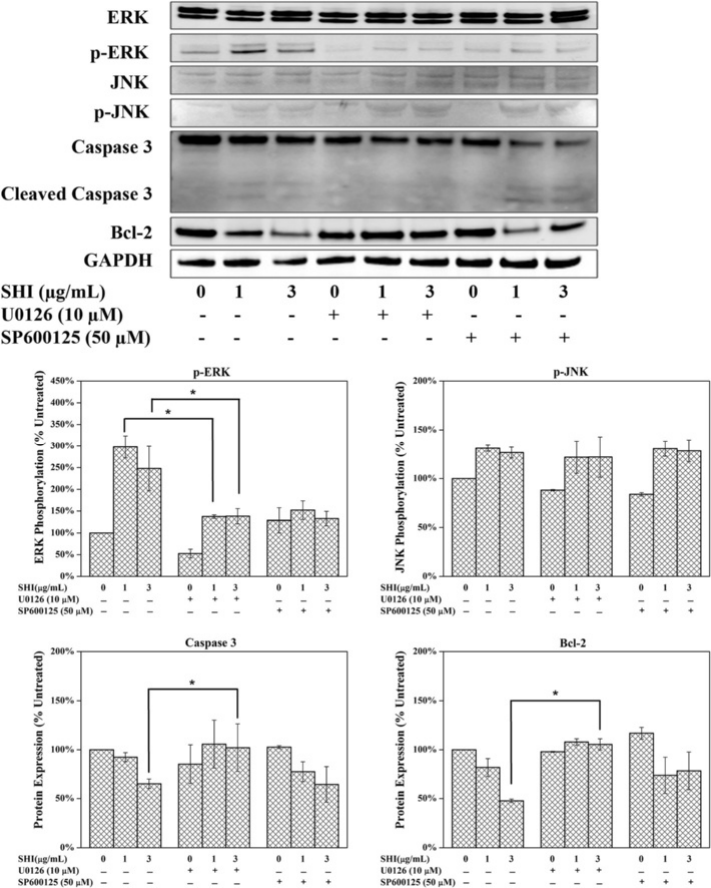
Link between MAPK and intrinsic apoptosis signalling pathway. HSF were treated with SHI with or without U0126 (10 μM) or SP600125 (50 μM) for 24 h. Expression of protein was measured using the Odyssey Infrared Imaging system. All experiments were performed 3 times using cells from 3 patients. Triplicate treatments were assessed in cells from each patient. The data was pooled as the percentage of the untreated control. Representative images of the western blots are presented. Quantitative data were pooled from experiments using cells 3 different patients. *p < 0.05

As described above, we demonstrated that SHI up-regulates p-ERK and p-JNK and down-regulates p-p38, caspase 3 and Bcl-2 expression in Kc and HSF in a dose-dependent manner. Reports in the literature indicate that the MAPK proteins play roles in regulating cell apoptosis processes [22, 23]. To investigate the roles of MAPK proteins in SHI-induced apoptosis, the p-ERK inhibitor U0126 and the p-JNK inhibitor SP600125 were used to block the phosphorylation of ERK and JNK in HSF when treated with SHI. The results of this analysis (Fig. 4) indicated that U0126 significantly inhibits SHI-induced up-regulation of p-ERK in HSF at 24 h. When HSF were treated with both SHI (1 and 3 μg/mL) and U0126, there was no reduction in Bcl-2 and cleaved caspase 3 was observed, indicating that the blockage of p-ERK interrupts SHI-induced down-regulation of Bcl-2 and cleavage of caspase 3. However, reductions in Bcl-2 and cleavage of caspase 3 were detected when HSF were treated with both SHI and SP600125, indicating that blockage of p-JNK has no effect on SHI-induced apoptosis in HSF.

### Effects of SHI on NF-κB signalling pathway

**Fig. 5:**
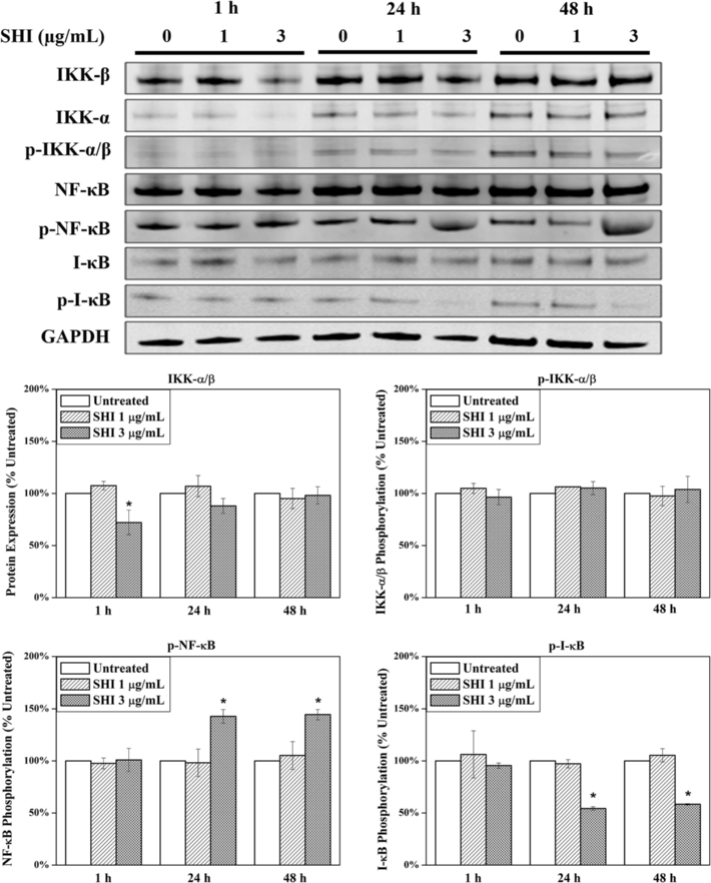
Effects of SHI on NF-κB signalling pathway. HSF were treated with SHI (0, 1 and 3 μg/mL) for 1, 24 or 48 h. Expression of protein was measured using the Odyssey Infrared Imaging system. All experiments were performed 3 times using cells from three patients. Triplicate treatments were assessed in cells from each patient. The data was pooled as the percentage of the untreated control. Representative images of the western blots are presented. Quantitative data were pooled from experiments using cells 3 different patients. *p < 0.05 versus the untreated control

A large number of studies have reported that nuclear factor-kappaB (NF-κB) plays essential roles in regulating inflammation and apoptosis [24, 25]. NF-κB normally binds with its inhibitor I-κB in an inactive state in the cytoplasm [26]. Phosphorylation of I-κB kinase-α/β (IKK-α/β) leads to the phosphorylation and subsequently degradation of I-κB, thus inducing the phosphorylation of NF-κB [27]. Activated (phosphorylated) NF-κB translocates into the nucleus and then regulates the transcription of its target genes, such as IL-1 and BCL2 [28]. As illustrated in Fig. 5, expression of total IKK-α/β in HSF was attenuated when exposed to SHI at 3 μg/mL for 1 h compared to the control (*p* < 0.05). However, the expression of p-IKK-α/β remained at the same level compared to the control. Down-regulation of p-I-κB and up-regulation of p-NF-κB were observed in HSF when exposed to SHI at 3 μg/mL for 24 and 48 h compared to the control. This data indicates that SHI at 3 μg/mL activates the NF-κB signalling pathway in HSF.

### Effects of SHI on cell gene expression

**Fig. 6:**
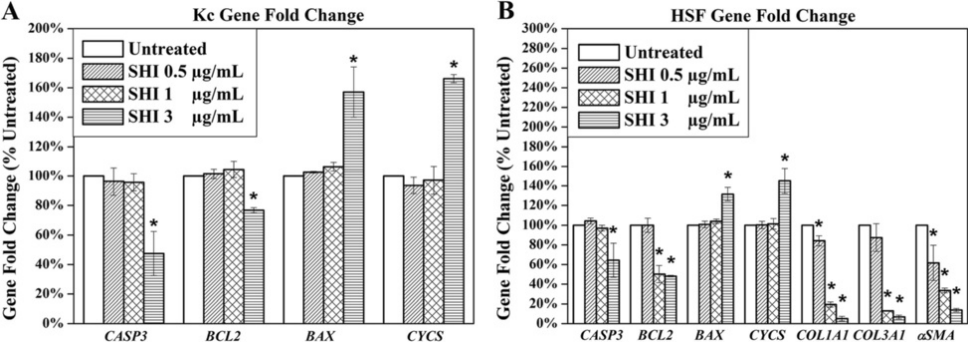
Effects of SHI on cell gene expression. **a** Gene Expression in Kc; **b** Gene expression in HSF. Kc and HSF were treated with various concentrations of SHI for 24 h and then total RNA was collected. After RNA extraction, first strand cDNA was synthesized. The cDNA sample was then amplified using qRT-PCR. The expression of the target gene was first normalized to GAPDH and then further converted to the percentage of the untreated control. Error bars indicate mean +/− SEM (*n* = 3). *p < 0.05 versus the untreated control. Statistical analysis was performed using One-way ANOVA and Tukey’s post-hoc test

In addition to *CASP3* and *BCL2*, other apoptosis-related genes, namely *BAX* and *CYCS* [21] in both Kc and HSF, and collagen production-related genes, including *COL1A1*, *COL3A1* and alpha-Smooth Muscle Actin (*αSMA*) [29] in HSF, were investigated using qRT-PCR (Fig. 6). As shown in Fig. 6a, SHI at 3 μg/mL significantly attenuated Kc *CASP3* and *BCL2* gene expression, while increasing *BAX* and *CYCS* gene expression when compared to the untreated control (*p* < 0.05). However, no effect at either 0.5 or 1 μg/mL SHI was observed on Kc gene expression. SHI at 3 μg/mL significantly down-regulated *CASP3*, *BCL2*, *COL1A1*, *COL3A1* and *αSMA* and up-regulated *BAX* and *CYCS* expression in HSF compared to the untreated control (*p* < 0.05) (Fig. 6b). In addition, decreases in *BCL2*, *COL1A1*, *COL3A1* and *αSMA* were observed in HSF after exposure to SHI at 1 μg/mL compared to the untreated control (*p* < 0.05). Furthermore, *COL1A1* and *αSMA* gene expression were below the untreated control (*p* < 0.05) in HSF exposed to 0.5 μg/mL SHI. These data indicate that SHI regulates the expression of the indicated apoptosis- and collagen production-related genes in a dose-dependent manner. Interestingly, SHI at 0.5 μg/mL inhibits *COL1A1* and *αSMA* gene expression without affecting apoptosis-related gene expression. RT-PCR has also been performed to detect the gene expression of *COL1A1*, *COL3A1* and *αSMA* in Kc, however, the data obtained indicated that these three genes are “Undetectable”.

### SHI reduces TGF-β1 expression

**Fig. 7:**
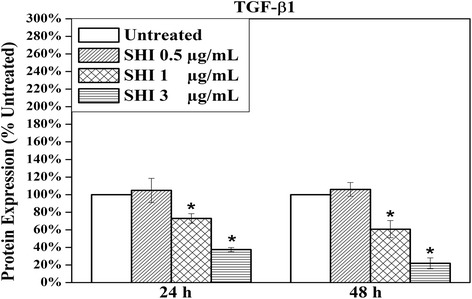
Effects of SHI on the expression of total TGF-β1 in Kc-HSF co-culture “conditioned” media. Cells were treated with SHI for 24 and 48 h and then the “conditioned” media were collected. The concentration of TGF-β1 was measured according to the standard curve provided with the assay kit. The expression of TGF-β1 was then further converted to the percentage of the untreated control. Error bars indicate mean +/− SEM (*n* = 3). *p < 0.05 versus the untreated control. Statistical analysis was performed using One-way ANOVA and Tukey’s post-hoc test

TGF-β1, produced by both Kc and HSF [30], has been broadly reported to play vital roles in wound healing processes [31]. Of particular relevance, the over-abundant expression of TGF-β1 in wound healing has been reported to result in the formation of HS [32]. The effect of SHI on total TGF-β1 expression in Kc-HSF co-culture was therefore investigated using an ELISA assay (Fig. 7). Our results suggest that total TGF-β1 expression is significantly reduced after exposed to SHI at 1 and 3 μg/mL for either 24 or 48 h compared to the untreated control (*p* < 0.05).

## Discussion

Hypertrophic scarring remains a significant problem in the clinic and the pathological mechanisms of HS are poorly understood [6]. This is complicated by the fact that the formation of HS may result from dysfunction in any stage of the wound healing process [3].

Numerous reports have indicated that reduced apoptosis of fibroblasts at the end of wound healing results in an increased number of active fibroblasts, and thereby induces the formation of HS [2]. Indeed, it has been reported that HS have a 4-fold decrease in cellular apoptosis compared with that found in normal wound scars [33]. Consistent with our preliminary data, SHI has been found in the studies reported herein to inhibit HSF proliferation and induce HSF apoptosis in a dose-dependent manner, even in the presence of co-cultured Kc. Of relevance, ERK1/2, JNK1/2 and p38α/β have been widely demonstrated to play vital roles in the regulation of cell proliferation and apoptosis [18, 34, 35]. Thus, it has been demonstrated that MAPKs regulate cell proliferation via mediating cell cycle progression [36]. In quiescent cells, ERK1/2 are mostly located in the cytoplasm, however, ERK1/2 accumulates in the nucleus once extracellular stimulation occurs [37]. ERK1/2 then phosphorylates c-Fos protein and allows c-Fos to bind with c-Jun to form an activator protein one (AP-1) complex [38, 39]. AP-1, in turn, induces the expression of cyclin D1, which stimulates the transition of the G1 to S phase of the cell cycle by interacting with cyclin-dependent kinases [40]. Similarly, JNK1/2 have been found to play essential roles in cell proliferation by supporting the formation of the AP-1 complex and transcription of cell cycle-related proteins such as cyclin D1 [41]. In addition, p-38 has been shown to regulate the cell cycle at the G_1_ to S and G_2_ to M transition points [42].

MAPKs have also been reported to participate in the regulation of apoptosis. For example, ERK1/2 regulates cellular apoptosis by affecting the expression of caspases [23]. Lee et al. demonstrated that p-ERK inhibitor significantly prevents Perfluorohexane Sulfonate-induced apoptosis in PC12 pheochromocytoma cells [22]. JNK1/2 on the other hand have been found to play an anti-apoptotic role by blocking the release of cytochrome *c* in UV treated fibroblasts [43]. In addition, studies by Qiu et al. reported that phosphorylation of p38α/β induces apoptosis via increasing oxidative stress and caspase-3 activity [44].

**Fig. 8:**
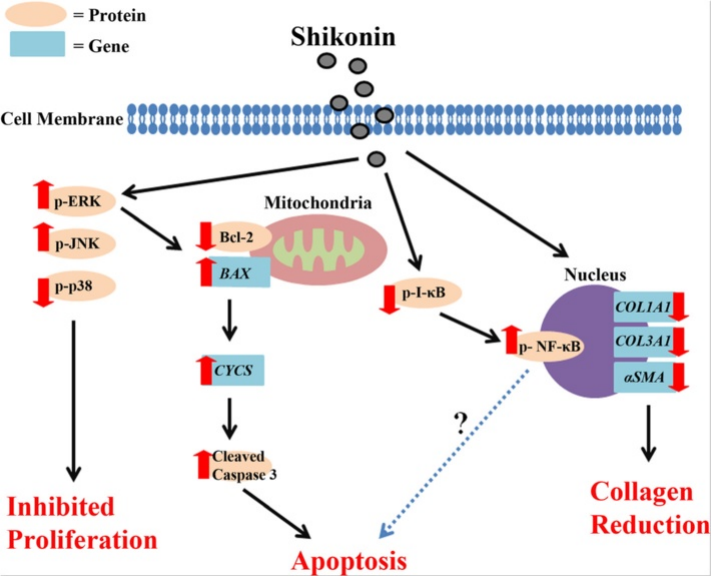
Potential signalling pathways triggered when Kc and HSF are treated with SHI. “” = activation or up-regulation; “” = inhibition or down-regulation

In light of this significant body of evidence, we examined which signal transduction pathways were involved in mediating the effects of SHI. The data reported here indicates that SHI-induced reduction of cell proliferation in Kc and HSF may be triggered via the ERK1/2, JNK1/2 and p38α/β signalling pathways. In addition, our results show that SHI induces apoptosis by differentially regulating the expression of Bcl-2, Bax, Cytochrome *c* and caspase 3. Moreover, our data demonstrated that p-ERK is an up-stream regulator mediating the expression of Bcl-2 and caspase 3. It has been reported that reduced Bcl-2 and increased Bax expression leads to the release of Cytochrome *c* from the mitochondria to the cytoplasm [45, 46]. Cytochrome *c* then combines with Apoptotic protease activating factor 1(Apaf-1), which further activates caspase 3 to trigger cell apoptosis [47]. These putative mechanisms are schematically summarised in Fig. 8.

NF-κB is another essential signalling pathway involved in regulating cell apoptosis and inflammation [24, 25]. NF-κB is activated (phosphorylated) via the phosphorylation of IKK-α/β and degradation (phosphorylation) of I-κB [27]. It has been reported that SHI induces apoptosis in the cancer cell lines PANC-1, BxPC-3 and AsPC-1 by suppressing the phosphorylation of NF-κB [48]. However, Kasibhatla et al. demonstrated that inhibition of NF-κB activity leads to reduced apoptosis in hybridoma cells [49]. Moreover, evidence indicates that activation of NF-κB leads to increased transcription of anti-apoptotic genes, such as Bcl-2, thus preventing cells from apoptosis [50]. Conversely, other studies suggest that activation of NF-κB is accompanied with decreased Bcl-2 expression [51]. Our studies, however, indicate degradation of I-κB and phosphorylation of NF-κB in HSF when treated with SHI, suggesting a potential role for NF-κB in SHI-induced apoptosis. Clearly, additional studies are required to determine the exact roles of NF-κB in this SHI-induced apoptosis in HSF; this could for example be provided through the use of luciferase assays to determine NF-κB-regulated gene transcription in HSF when treated with SHI.

Kc also play important roles in wound healing and HS formation. Indeed it has been reported that if wound closure takes more than 21 days, then the probability of the wound developing into a HS is more than 78 % [52]. Although our results show that SHI also inhibits Kc proliferation and induces Kc apoptosis through the same signalling pathways as in HSF, higher concentration of SHI (3 μg/mL) are required to activate those signalling pathways in Kc compared to that observed in HSF. In other words, SHI can inhibit HSF proliferation and induce HSF apoptosis without affecting Kc function when applied at specific concentrations (1 μg/mL). These results further support the potential use of SHI as a novel scar treatment since they suggest that SHI may be able to be applied to inhibit excessive fibroblast proliferation during HS formation while having little effect on the re-epithelialization function of Kc.

Another important factor underlying HS formation is the over-abundant amount of collagen produced by fibroblasts [53]. In normal wound healing the high level of collagen synthesis present in the early stages decreases and returns to normal tissue levels as the wound closes [5]. Deficient degradation or excessive production of collagen may therefore result in HS [7]. The data reported here indicates that SHI significantly down-regulates *COL1A1* and *COLA3A1* gene expression. In addition, *αSMA*, a marker of myo-fibroblasts (a highly proliferative fibroblast type that produces more collagen than normal fibroblasts) [29], was also attenuated after SHI treatment. Interestingly, 0.5 μg/mL of SHI decreases *COL1A1* and *αSMA* gene expression in HSF, while no effect of SHI on HSF proliferation or apoptosis was observed at this concentration. These data suggest that it might be possible to deliver SHI at concentrations that inhibit collagen production by fibroblasts without affecting cell proliferation.

Cytokines, such as TGF-β1 [54], Platelet-derived Growth Factor (PDGF) [55] and Vascular Endothelial Growth Factor (VEGF) [56], are also involved in wound healing and HS formation processes. TGF-β1, a cytokine secreted by both Kc and fibroblasts during wound healing, has been reported to be up-regulated in HS tissues [57, 58]. Furthermore, TGF-β1 from Kc stimulates fibroblasts to become myo-fibroblasts [59]. In contrast, TGF-β1 secreted by fibroblasts induces Kc to return to an inactive state at the end stage of wound healing [60]. Our results suggest that SHI attenuates the expression of TGF-β1 in Kc-HSF co-culture conditioned medium, further supporting the concept of SHI as a potential therapy for HS. While we also employed ELISA assays to examine the expression of PDGF and VEGF, the concentration of PDGF and VEGF in the Transwell® “conditioned” medium was too low to be detected using ELISA. The effect of SHI on those cytokines needs to be further evaluated using different approaches, such as animal scar models, in future studies.

TGF-β1 has also been reported to play either anti-apoptotic or pro-apoptosis functions depending on the type of cell [61], however, the exact mechanisms of TGF-β1-induced apoptosis are still elusive. Jang et al. demonstrated that TGF-β1 induces apoptosis in the human hepatoma cell line Hep3B via activating the death-associated protein kinase (DAP-kinase) [62]. However, Huang et al. reported that TGF-β1 prevents the human lung carcinoma cell line A549 from serum deprivation-induced apoptosis by reducing p-JNK expression [63]. Most relevant to wound healing, Karimizadeh et al. suggested that TGF-β1 prevents dermal fibroblasts from apoptosis [64]. To reveal the link between TGF-β1 reduction and SHI-induced apoptosis, further studies using mono-cultured keratinocytes or fibroblasts are required.

In conclusion, the excessive proliferation of fibroblasts, over-abundant production of collagen and delayed Kc function are three essential aspects linked to HS formation. SHI, an active component extracted from the Chinese herb *Radix Arnebiae*, has been demonstrated here for the first time to inhibit HSF proliferation and induce apoptosis without affecting Kc function at select concentrations. In addition, SHI has been demonstrated to reduce collagen production in HSF and attenuate TGF-β1 expression in Kc-HSF co-culture “conditioned” medium.

## Conclusions

The data generated from this study not only improves our understanding of the bioactivities of SHI in a new range of cells, but also suggests that SHI as a potential treatment for HS warrants further investigation.

## Abbreviations

αSMA: Alpha-smooth muscle actin
Bax: Bcl-2-associated X protein
BAX: Bax gene
Bcl-2: B-cell lymphoma 2
BCL2: Bcl-2 gene
CASP3: Caspase 3 gene
Caspases: Cysteine-dependent aspartate-directed proteases
COL1A1: Collagen I gene
COL3A1: Collagen III gene
CYCS: Cytochrome *c* gene
DAPI: 4′, 6-diamidino-2-phenylindole
DMEM: Dulbecco’s modified eagle’s medium
DMSO: Dimethyl sulfoxide
ERK1/2: Extracellular signal-regulated kinase 1 and 2
FCS: Fetal calf serum
hs: hypertrophic scars
HSF: Hypertrophic scar-derived human skin fibroblasts
JNK1/2: c-Jun N-terminal kinase 1 and 2
Kc: Human skin keratinocytes
MAPK: Mitogen-activated protein kinases
p38α/β: Mitogen-activated protein kinase 14 α and β
qRT-PCR: Quantitative reverse transcriptase polymerase chain reaction
SDS-PAGE: Sodium dodecyl sulphate polyacrylamide gel electrophoresis
SEM: Standard error of the mean
SHI: Shikonin
TGF-β1: Transforming growth factor beta 1
TUNEL: Terminal deoxynucleotidyl transferase dUTP nick end labelling
